# Global trends and thematic clusters in healthcare professional education, medical errors, and patient safety: a bibliometric analysis, 2000–2026

**DOI:** 10.3389/fmed.2026.1892822

**Published:** 2026-09-03

**Authors:** Birgit Hladschik-Kermer, Olena Litvinova, Michel-Edwar Mickael, Maima Matin, Ahmed Fatimi, Anass Belbachir, Mairim Russo Serafini, Maria Kletecka-Pulker, Atanas G. Atanasov, Thomas Wochele-Thoma

**Affiliations:** 1 Unit Medical Psychology, Department of Public Mental Health, Center for Public Health, Medical University of Vienna, Vienna, Austria; 2 Ludwig Boltzmann Institute Digital Health and Patient Safety, Medical University of Vienna, Vienna, Austria; 3 Department of Management, Marketing and Quality Assurance in Pharmacy, National University of Pharmacy of the Ministry of Health of Ukraine, Kharkiv, Ukraine; 4 Department of Biotechnology and Nutrigenomics, Institute of Genetics and Animal Biotechnology of the Polish Academy of Sciences, Magdalenka, Poland; 5 Chemical Science and Engineering Research Team, Polydisciplinary Faculty of Beni Mellal, Sultan Moulay Slimane University, Beni Mellal, Morocco; 6 Regenerative Medicine Center, Mohammed VI University Hospital, Marrakech, Morocco; 7 Faculty of Medicine and Pharmacy, Cadi Ayyad University, Marrakech, Morocco; 8 Postgraduate Program in Health Sciences, Federal University of Sergipe, Aracaju, Sergipe, Brazil; 9 Department of Pharmacy, Federal University of Sergipe, São Cristovão, Sergipe, Brazil; 10 Institute for Ethics and Law in Medicine, University of Vienna, Vienna, Austria; 11 Department of Biochemistry, Saveetha Medical College and Hospital, Saveetha Institute of Medical and Technical Sciences, Chennai, Tamil Nadu, India

**Keywords:** burnout, digital health, healthcare professional education, interprofessional education, medical errors, mental health, patient safety, simulation-based training

## Abstract

**Background:**

In the context of the active implementation of the World Health Organization’s Global Patient Safety Action Plan, there is a growing need for a comprehensive synthesis of scientific evidence reflecting the development of research in the field of healthcare professional education.

**Objective:**

This study aims to conduct a bibliometric analysis of scientific publications addressing the relationship between healthcare professional education, medical errors, and patient safety over the period 2000–2026, based on the Web of Science and Scopus databases, to identify global trends, stages of development, and key thematic clusters of research in the digital era.

**Methods:**

The analysis was based on publications that met the search criteria and were indexed in the Web of Science and Scopus databases (2000–2026, as of April 27, 2026). Analytical tools provided by the databases, the bibliometrix R package, and VOSviewer software were used. Scientific productivity, citation metrics, contributions of leading journals and research institutions, keyword bursts and co-occurrence, as well as thematic structure, were analyzed.

**Results:**

A total of 17,457 publications that met the inclusion criteria were included in the analysis. A sustained increase in publication activity was identified. Four stages of development were defined: formation (2000–2008), rapid growth (2009–2014), thematic diversification (2015–2018), and interdisciplinary integration (2019–2026). Cluster analysis revealed four key research areas: educational strategies and competency development; healthcare systems and quality management; human factors and the epidemiology of medical errors; and medication safety. An increase in the number of publications devoted to simulation-based learning, interdisciplinary education and digital technologies was identified.

**Conclusion:**

A steady increase in publication activity, expansion of thematic areas, and strengthening of the interdisciplinary nature of research in the fields of medical education, medical errors, and patient safety were identified. This reflects the growing attention of the scientific community to issues considered among the WHO’s key patient safety priorities. The obtained results allow us to identify promising areas for further research, including the development of clinically relevant indicators linking educational interventions with patient safety outcomes, further exploration of educational programs aimed at supporting mental health, well-being, and preventing burnout in healthcare workers, and exploring the application of new technologies, including artificial intelligence, in medical education and patient safety.

## 1 Introduction

The use of medical interventions inevitably involves not only therapeutic benefits but also the risk of adverse events and medical errors, making patient safety one of the key priorities of modern healthcare. According to the World Health Organization’s Global Action Plan for 2021–2030, a priority area is the systematic integration of patient safety principles into healthcare professional education, including the development of educational standards, the assessment of competencies, and the creation of a safe clinical environment (1).

Despite the efforts being made, preventable medical errors continue to be a significant cause of adverse outcomes and substantial economic losses (2). Against a backdrop of increasingly complex clinical decisions and the digital transformation of healthcare, the level of training, clinical reasoning, and professional competence of healthcare professionals is becoming crucial. Recent research confirms the multifactorial nature of medical errors, in which knowledge gaps, cognitive biases, and decision-making patterns play a key role (3).

In response to these challenges, healthcare professional education is undergoing a transformation toward competency-based models that incorporate simulation-based learning, interprofessional collaboration and the use of digital technologies (4–6). The development of such approaches, based on simulation-based training, artificial intelligence (AI), virtual and augmented reality, as well as online learning, significantly expands the scope for developing both technical and non-technical skills in a controlled and safe environment (7, 10). However, despite the significant potential of these technologies, their impact on real-world clinical outcomes and the reduction in the incidence of medical errors remains understudied.

Bibliometric studies have emerged as a powerful methodological approach for systematically mapping and analyzing the structure, evolution, and intellectual landscape of academic knowledge across diverse scientific disciplines and fields (11, 12). In recent years, there has been an increase in the number of bibliometric studies focusing on specific aspects of healthcare professional education and patient safety. Zhou L. et al. demonstrated a steady increase in research on patient safety education from 2000 to 2022, with a focus on curriculum content, teaching methods, and competency assessment (13). Similarly, Wang Y. et al. reported an expansion of the research focus on nursing students’ training in patient safety, including simulation-based learning and the use of standardized patients (14). Furthermore, Alblooshi A. S. et al. demonstrated a rapid growth in publications on simulation-based medical education from 2001 to 2024, highlighting a shift from technical skills training toward the development of non-technical competencies, as well as the integration of virtual reality, augmented reality, and artificial intelligence (15). However, existing studies generally examine these areas in isolation, which limits the understanding of their interrelationships and the systemic dynamics of their development.

In the context of the active implementation of the World Health Organization’s Global Patient Safety Action Plan, there is a growing need for a comprehensive analysis of the research landscape, enabling an assessment of the alignment of current research in healthcare professional education with global health priorities and its contribution to achieving long-term goals.

This study aims to conduct a bibliometric analysis of scientific publications addressing the relationship between healthcare professional education, medical errors, and patient safety over the period 2000–2026, based on the Web of Science and Scopus databases, to identify global trends, stages of development, and key thematic clusters of research in the digital era.

## 2 Methods

Bibliometric analysis included quantitative, structural, and thematic assessment of scientific publications addressing the relationship between healthcare professional education, medical errors, and patient safety.

### 2.1 Databases and search strategy

The scientific publication search was conducted on 27 April 2026 in the Web of Science and Scopus databases. The use of both Web of Science and Scopus improved coverage compared with reliance on a single database and reduced the risk of missing relevant publications.

The search strategy was developed using a combination of key terms and Boolean operators (AND/OR) and included three conceptual blocks: educational approaches and teaching methods; medical errors, adverse events, and patient safety; and target groups, including trainees and healthcare professionals. The final search query was formulated as follows:

(“teaching*” OR “curriculum*” OR “curricula*” OR “educat*” OR “workshop*” OR “seminar*” OR “online course*” OR “eLearning*” OR “online learning*” OR “E-learning*” OR “electronic learning*” OR “computer-assisted learning*” OR “computer-mediated learning*” OR “computer-based learning*” OR “digital learning*” OR “simulation-based training*” OR “simulation-based learning*”)

AND

(“medical error*” OR “medication error*” OR “clinical error*” OR “diagnostic error*” OR “adverse event*” OR “adverse outcome*” OR “patient safety*” OR “patient harm*” OR “clinical incident*” OR “preventable harm*” OR “iatrogenic*”)

AND

(“student*” OR “resident*” OR “trainee*” OR “physician*” OR “doctor*” OR “nurse*” OR “clinician*” OR “healthcare professional*”)

The analysis covered the period from 1 January 2000 to 27 April 2026. The lower bound of the time frame was chosen in light of the publication “To Err Is Human: Building a Safer Health System,” which served as a key catalyst for research on patient safety (16).

The complete search strategies for each database, including search fields, search queries, and applied filters, are presented in Supplementary Table S1. Bibliographic records were exported from Web of Science in “Plain Text” format and from Scopus in “BibTeX” format and imported into bibliometrix (R). The standard bibliometrix workflow was used to merge data from both databases, detect and remove duplicates, and generate a single bibliographic dataset for subsequent analysis. To ensure accurate accounting of publication activity, different spelling variants of the names of the same institution were converted to a single name. The analysis of bibliographic indicators was performed using bibliometrix tools based on metadata exported from Web of Science and Scopus. To visualize the keyword co-occurrence network, the combined dataset generated in bibliometrix and imported into VOSviewer (version 1.6.18) was used. The analysis was performed for all keywords. The network included keywords that occurred at least 175 times. The remaining network construction parameters corresponded to the standard settings of the VOSviewer program.

### 2.2 Inclusion and exclusion criteria for publications

The publication search was conducted in the Web of Science and Scopus databases for the period 2000–2026. Only publications meeting the following criteria were included in the final analysis: publication period—2000–2026; document type—original articles and reviews; publication language — English.

The initial dataset comprised 14,872 records in Web of Science and 9,101 records in Scopus for the period 2000–2026. After applying time restrictions and filtering by document type and language, 13,502 and 7,395 records remained, respectively.

The combined dataset comprised 20,897 records. Duplicates were removed using the bibliometrix package in R (version 4.5.2; R Core Team, 2025) (17). As a result, 3,440 duplicates were excluded, leaving a final dataset of 17,457 unique publications.

**Figure 1:**
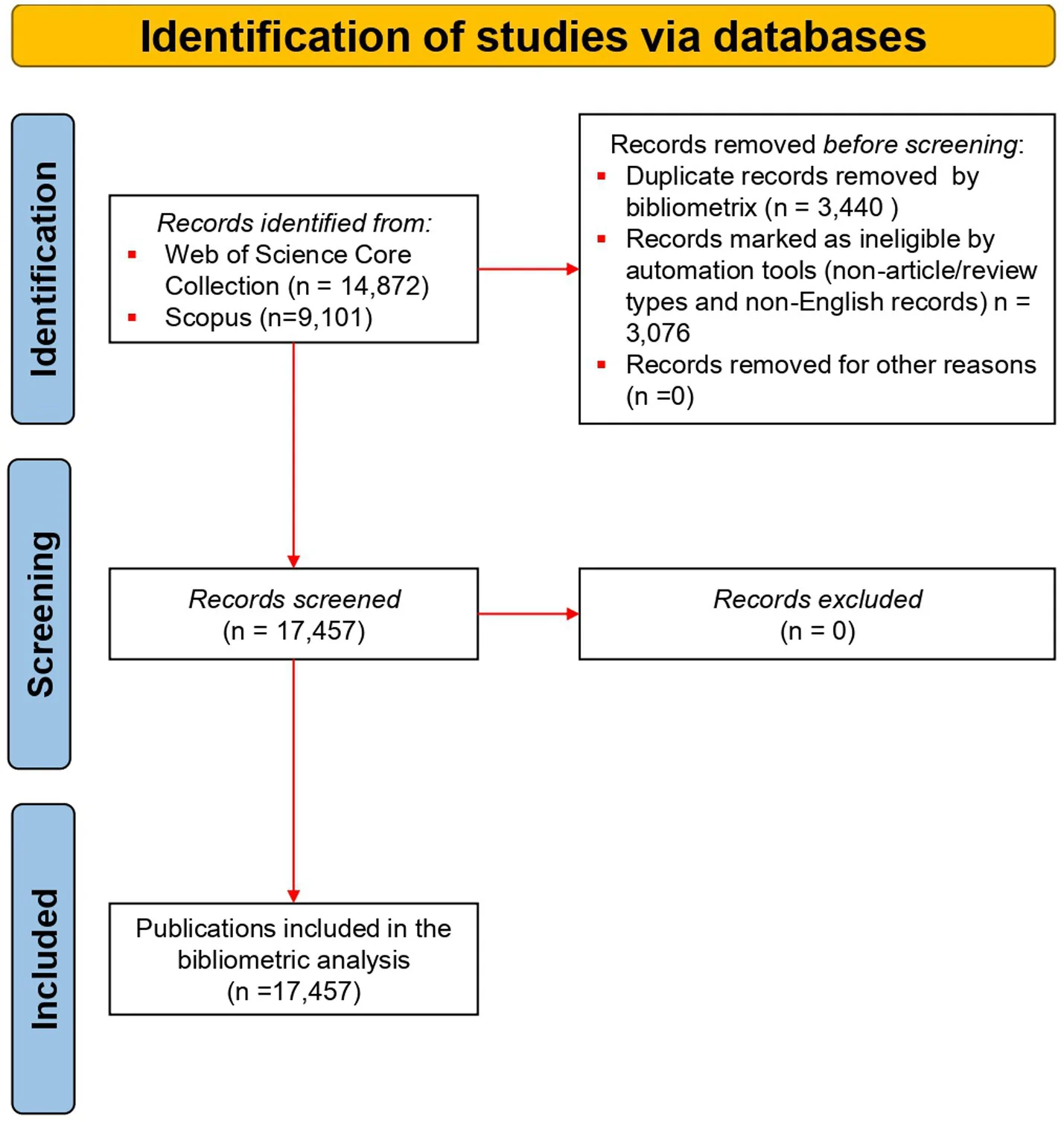
Publication selection scheme (PRISMA) for bibliometric analysis. This diagram illustrates the step-by-step process of identifying, automatically filtering, removing duplicates, and incorporating research papers into the final dataset, adapted for a bibliometric study.

The process of identification, automated filtering, removal of duplicates and final inclusion of publications was illustrated using a PRISMA diagram adapted for bibliometric research (Figure 1) (18).

In our study, we employ bibliometric analysis, which differs from traditional systematic reviews and meta-analyses, which involve detailed full-text screening of publications. According to widely recognized methodological works in the field of bibliometrics (19, 20), the primary objective of bibliometric analysis is the macro-level assessment of large datasets to identify structural, thematic and evolutionary trajectories in the development of a research field. In the present study, the relevance and integrity of the dataset were ensured through a carefully designed search syntax and selected keywords, refined database filters, and comprehensive data cleaning in accordance with standard bibliometric procedures (17). Bibliometric analysis complements traditional systematic reviews and meta-analyses, enabling a broader scientific landscape to be depicted while reducing subjectivity related to researcher-driven selection and interpretation (17).

However, manual screening of records can improve thematic accuracy by identifying publications that fall outside the scope of the research area (19). In light of this, and given that our search strategy was deliberately broad to support comprehensive bibliometric mapping, we conducted an additional assessment of dataset specificity to confirm the thematic relevance of the final corpus. This approach mirrors that used, for example, in a recent bibliometric study on extended reality technologies in healthcare professional education (21).

We conducted an automated term audit of all 17,457 records using the title, abstract, author keywords, and Keywords Plus. The audit assessed whether each record contained terms corresponding to the three conceptual blocks of the search strategy: education/training, patient safety/medical errors/adverse events, and healthcare professionals or learners.

To further assess the relevance of the dataset, a manual review of a random sample comprising 10% of the final corpus (1,746 records) was also conducted. The sample was proportionally distributed across four publication periods to ensure representativeness of the entire study period. Records were classified as directly relevant, content-related, or weak/possibly outside the scope depending on the extent to which the title and abstract explicitly linked healthcare professional education with patient safety topics. The audit was used solely to assess dataset specificity, and no records were excluded from the bibliometric dataset.

### 2.3 Bibliometric analysis

The bibliometric study was conducted using the bibliometrix package in R (version 4.5.2; R Core Team, 2025) and included an assessment of research productivity, the most productive journals, leading research organizations, and the citation rates of publications (17).

**Figure 2:**
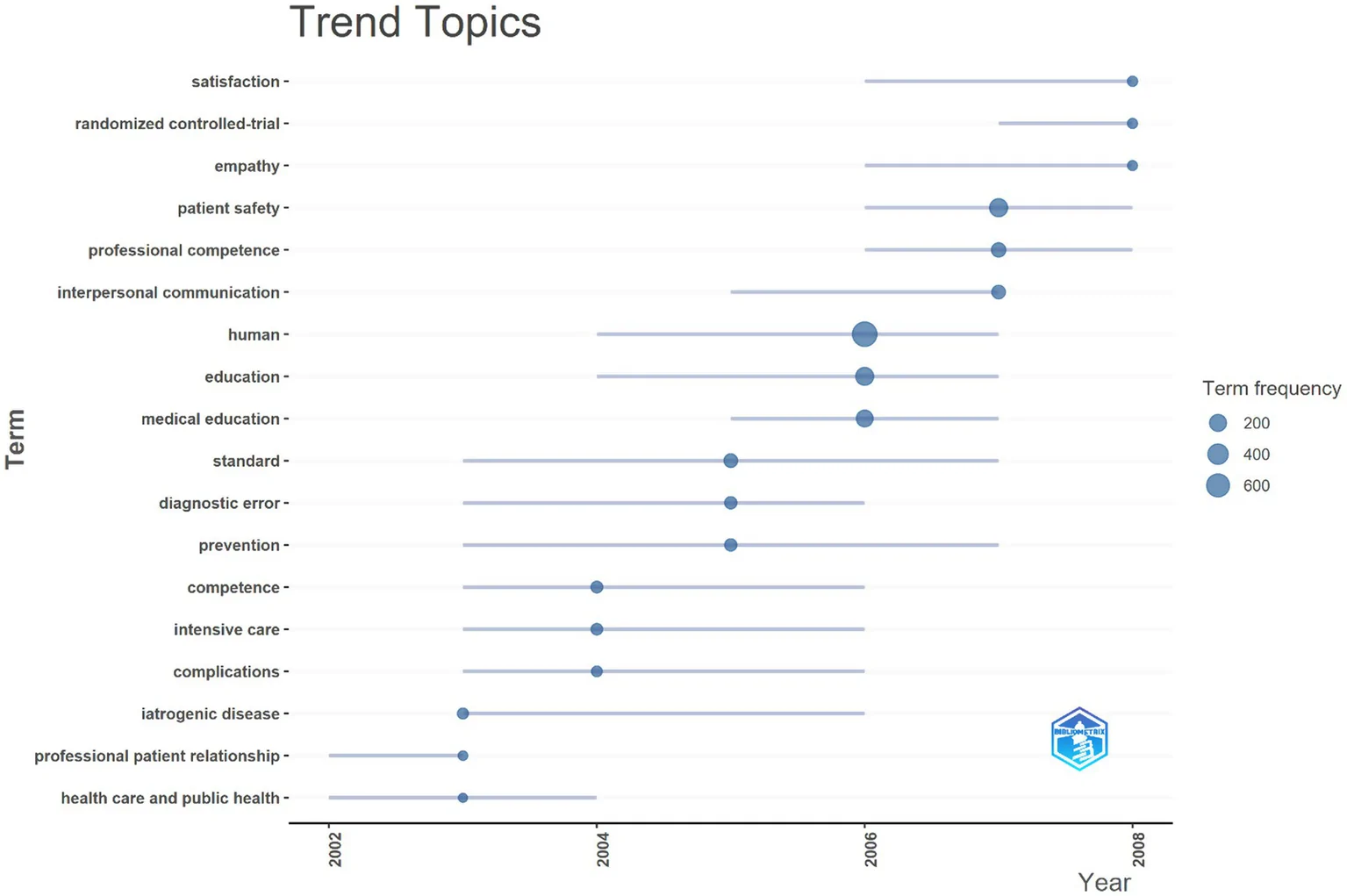
Keyword trends with peak bursts (≥5), bibliometrix, 2000–2008. The “Trend Topics” graph, generated using the Bibliometrix package, is designed to visualize the temporal evolution of keywords. The size of the points reflects the frequency of each keyword, while the horizontal lines indicate the time interval during which each keyword appeared in the dataset.

**Figure 3:**
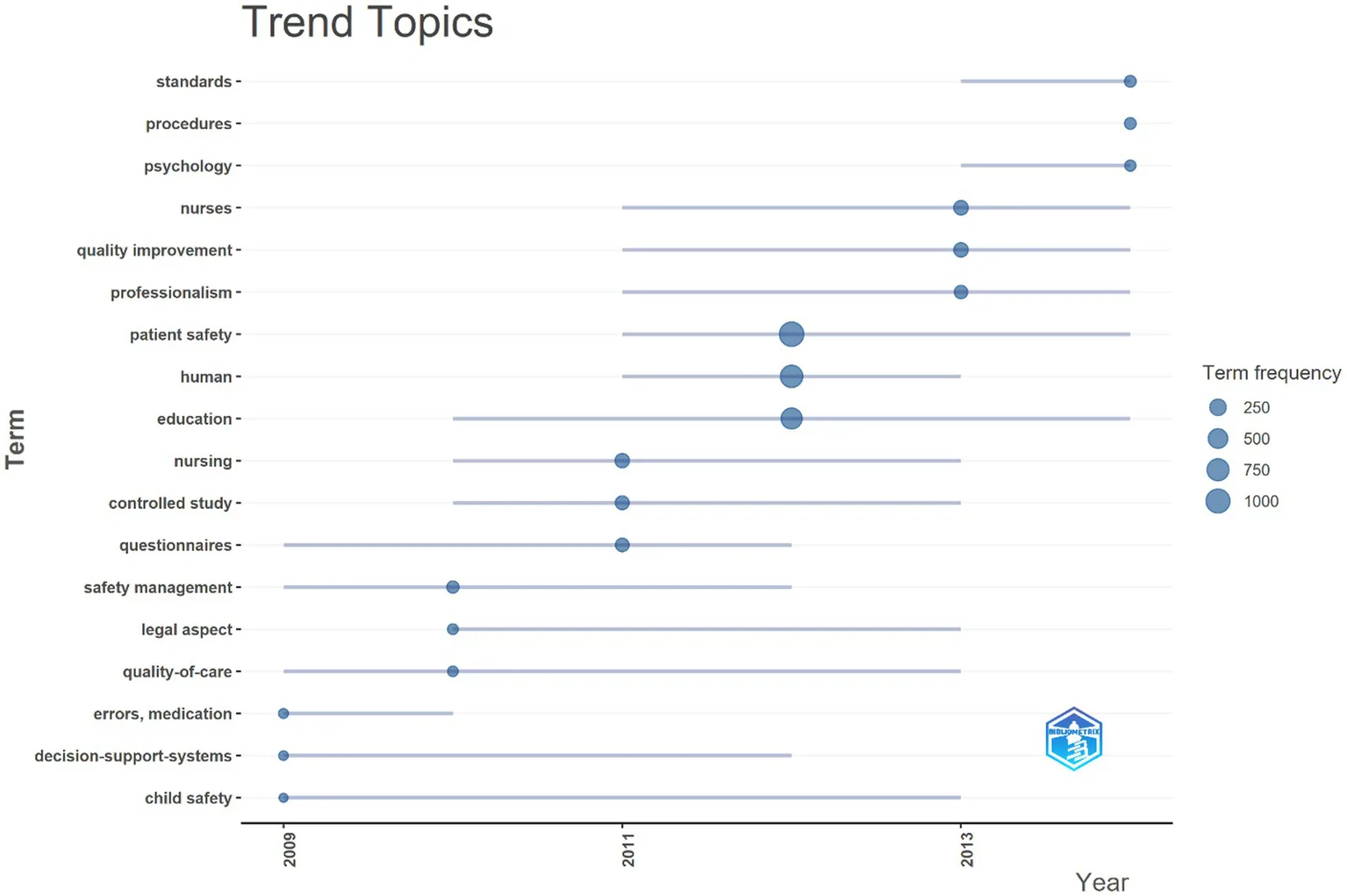
Keyword trends with peak bursts (≥5), Bibliometrix, 2009–2014. The “Trend Topics” graph, generated using the Bibliometrix package, is designed to visualize the temporal evolution of keywords. The size of the points reflects the frequency of each keyword, while the horizontal lines indicate the time interval during which each keyword appeared in the dataset.

**Figure 4:**
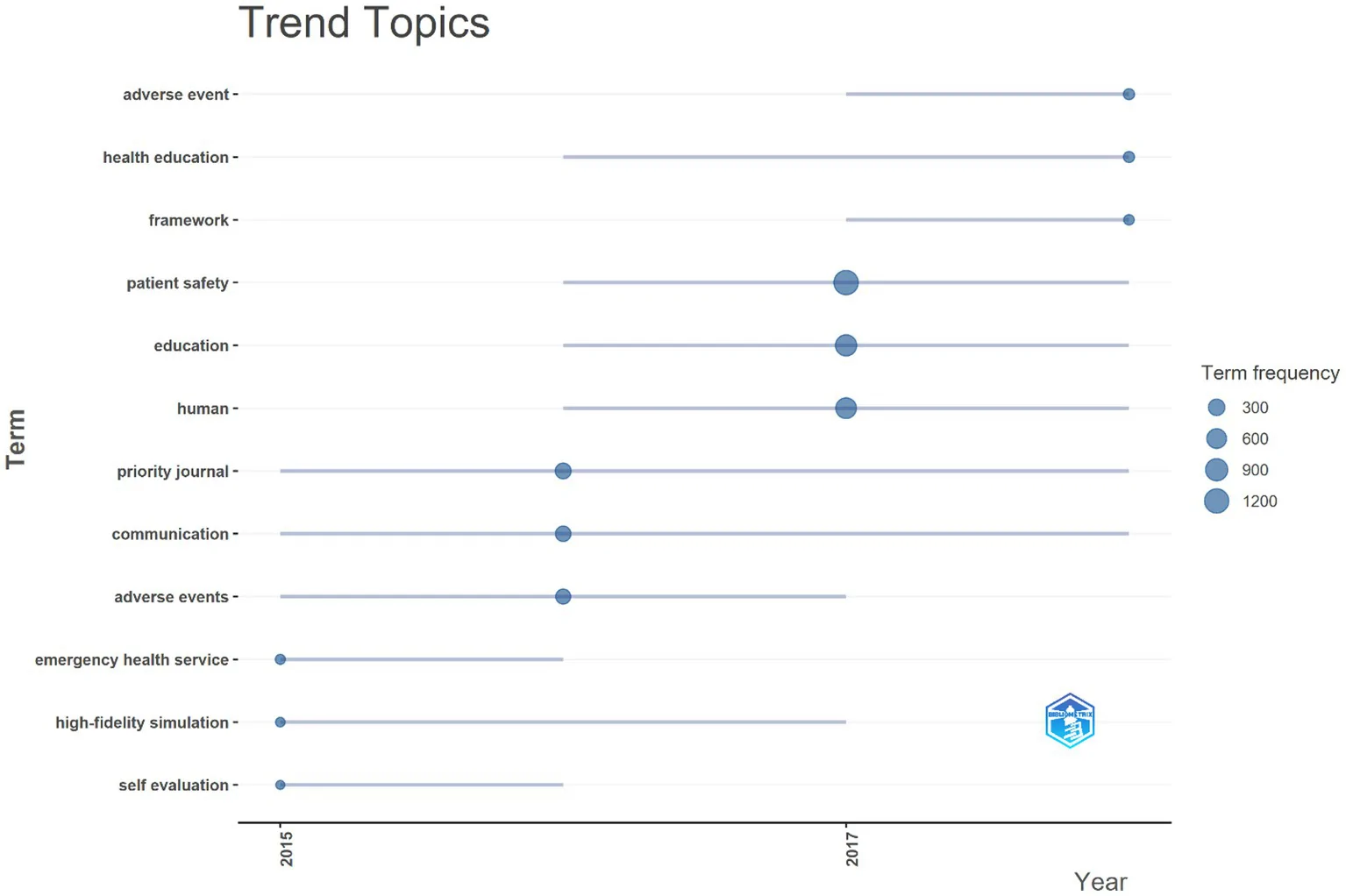
Keyword trends with peak bursts (≥5), bibliometrix, 2015–2018. The “Trend Topics” graph, generated using the Bibliometrix package, is designed to visualize the temporal evolution of keywords. The size of the points reflects the frequency of each keyword, while the horizontal lines indicate the time interval during which each keyword appeared in the dataset.

**Figure 5:**
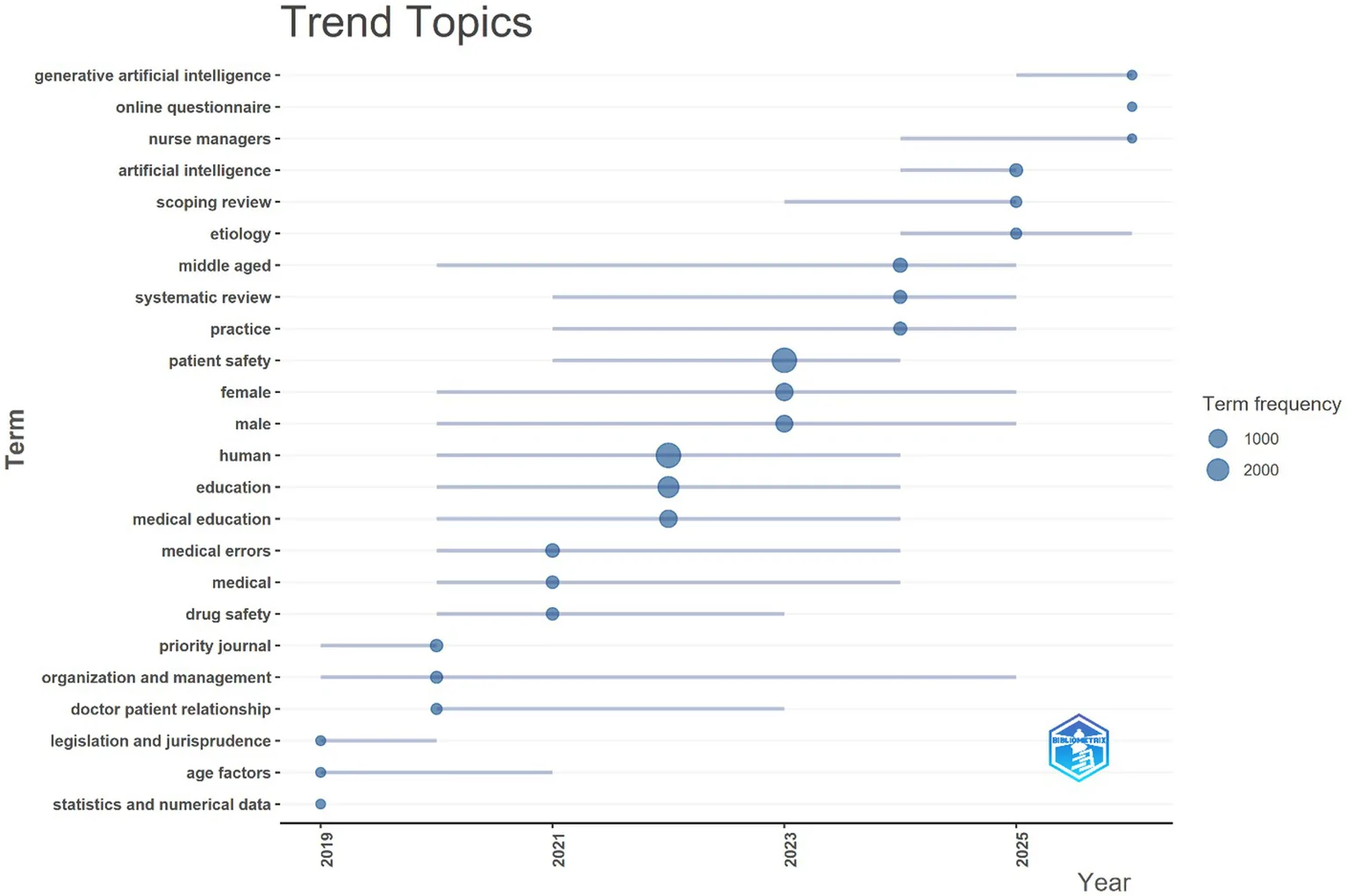
Keyword trends with peak bursts (≥5), Bibliometrix, 2019–2026. The “Trend Topics” graph, generated using the Bibliometrix package, is designed to visualize the temporal evolution of keywords. The size of the points reflects the frequency of each keyword, while the horizontal lines indicate the time interval during which each keyword appeared in the dataset.

To study the evolution of research fields, the trend topics approach was applied, allowing assessment of the temporal dynamics of keywords and identification of periods of heightened scientific interest, characterized by bursts in their frequency. The analysis included only terms with a frequency of ≥5, which reduced the influence of low-frequency and irrelevant terms (17). All frequency calculations, metadata filtering and automatic trend visualization were carried out in the R environment (v 4.5.2) (R Core Team, 2025) using the bibliometrix package (Figures 2–5 show the bibliometrix logo). Following this automated stage, the qualitative interpretation of the extracted keywords and the narrative description in the “Results” section were independently reviewed and validated by two authors (O. L. and A. G. A.), who possess expertise in the fields of healthcare professional education and patient safety. Any discrepancies in the interpretation of themes were resolved through joint discussion until a consensus was reached. These interpretations are presented as bibliometric findings and did not include a qualitative thematic analysis of the full texts of the articles and training programs.

### 2.4 Cluster analysis

VOSviewer software (version 1.6.18) was used to visualize the thematic mapping (22). The co-occurrence analysis of keywords was performed using a threshold of at least 175 mentions (≥1% of the total dataset), which ensured the stability and interpretability of the thematic clusters (22).

### 2.5 Analysis of temporal dynamics

The evolution of the research field was assessed on the basis of annual publication activity, including the absolute number of publications, growth rates, and trends. Based on structural changes in publication activity, four stages of development were identified: 2000–2008 — formation of the field (1,272 publications); 2009–2014 — accelerated growth (3,066 publications); 2015–2018 — thematic diversification (3,354 publications); 2019–2026 — interdisciplinary integration (9,765 publications).

The combination of bibliometrix and VOSviewer enabled a comprehensive multidimensional analysis, including quantitative assessment and thematic mapping of the development of the field under study.

## 3 Results

### 3.1 General bibliometric characteristics

The results of the study indicate a sustained growth in publication activity in the field of healthcare professional education, medical error prevention, and patient safety between 2000 and 2026.

A total of 17,457 unique English-language publications were included in the analysis, of which 15,009 (85.98%) were original research articles, and 2,448 (14.02%) were reviews.

The data obtained indicate that most publications are primary research papers. The significant proportion of review publications indicates the accumulation of research and the growing need for its systematization and synthesis.

The specificity assessment confirmed the thematic alignment of the corpus. The automated analysis demonstrated the presence of terms related to education/training in 100.00% of records, patient safety/errors in 99.68%, and healthcare professionals or learners in 98.57%. All three conceptual blocks were simultaneously present in 98.26% of records.

In the manual audit of a stratified random sample comprising 10% of the dataset (1,746 records), conducted by two independent authors, 1,394 records (79.84%) were classified as directly relevant, 337 (19.30%) as content-related, and 15 (0.86%) as weakly related or potentially outside the scope of the study. Overall, 1,731 of the 1,746 reviewed records (99.14%) were either directly relevant or related to the research topic.

**Figure 6:**
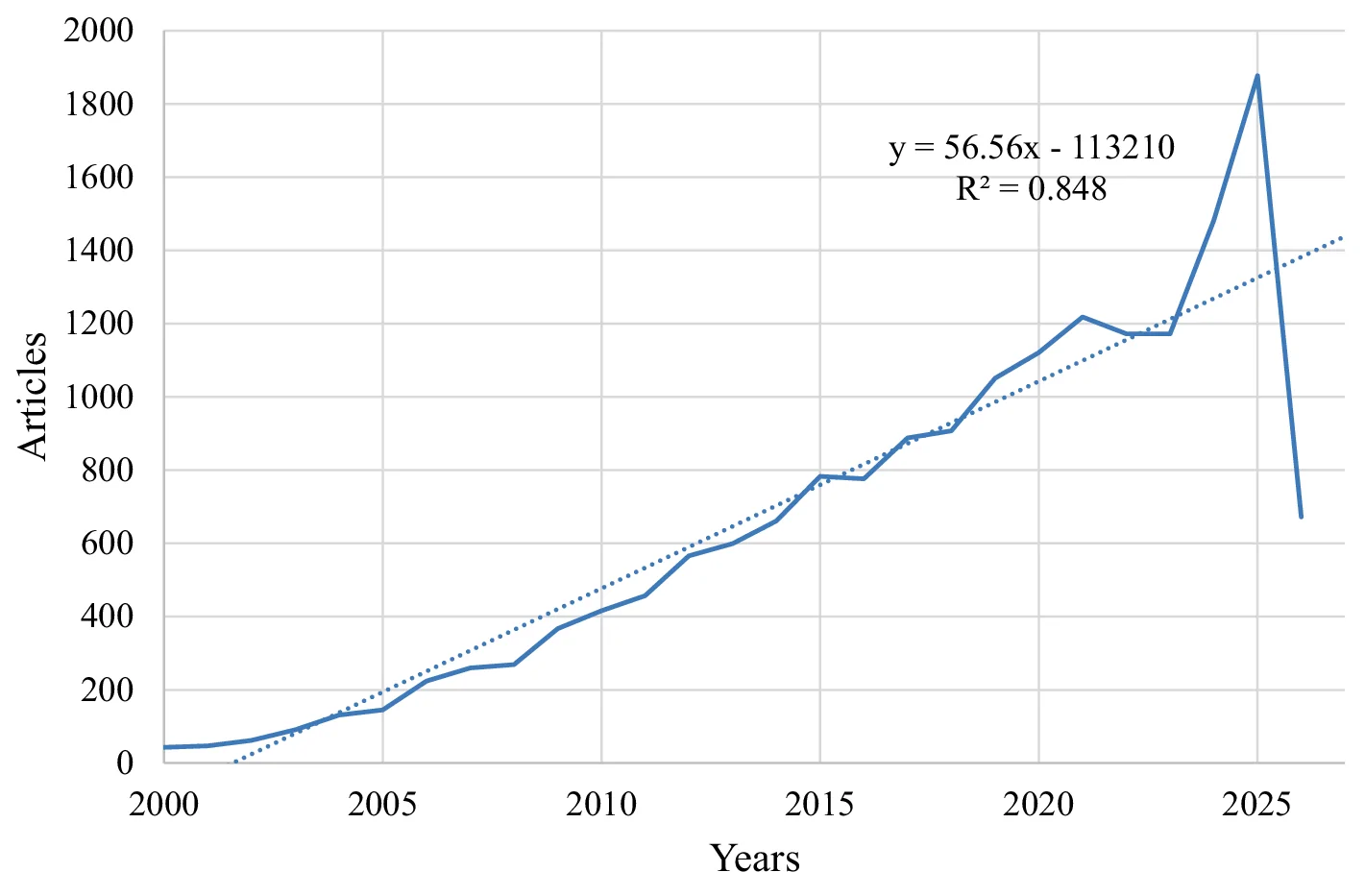
Annual publication dynamics.

The dynamics of annual publication activity showed steady growth from 43 publications in 2000 to 1,877 in 2025. The most pronounced increase was observed at the later stage of the field’s development (Figure 6).

**Figure 7:**
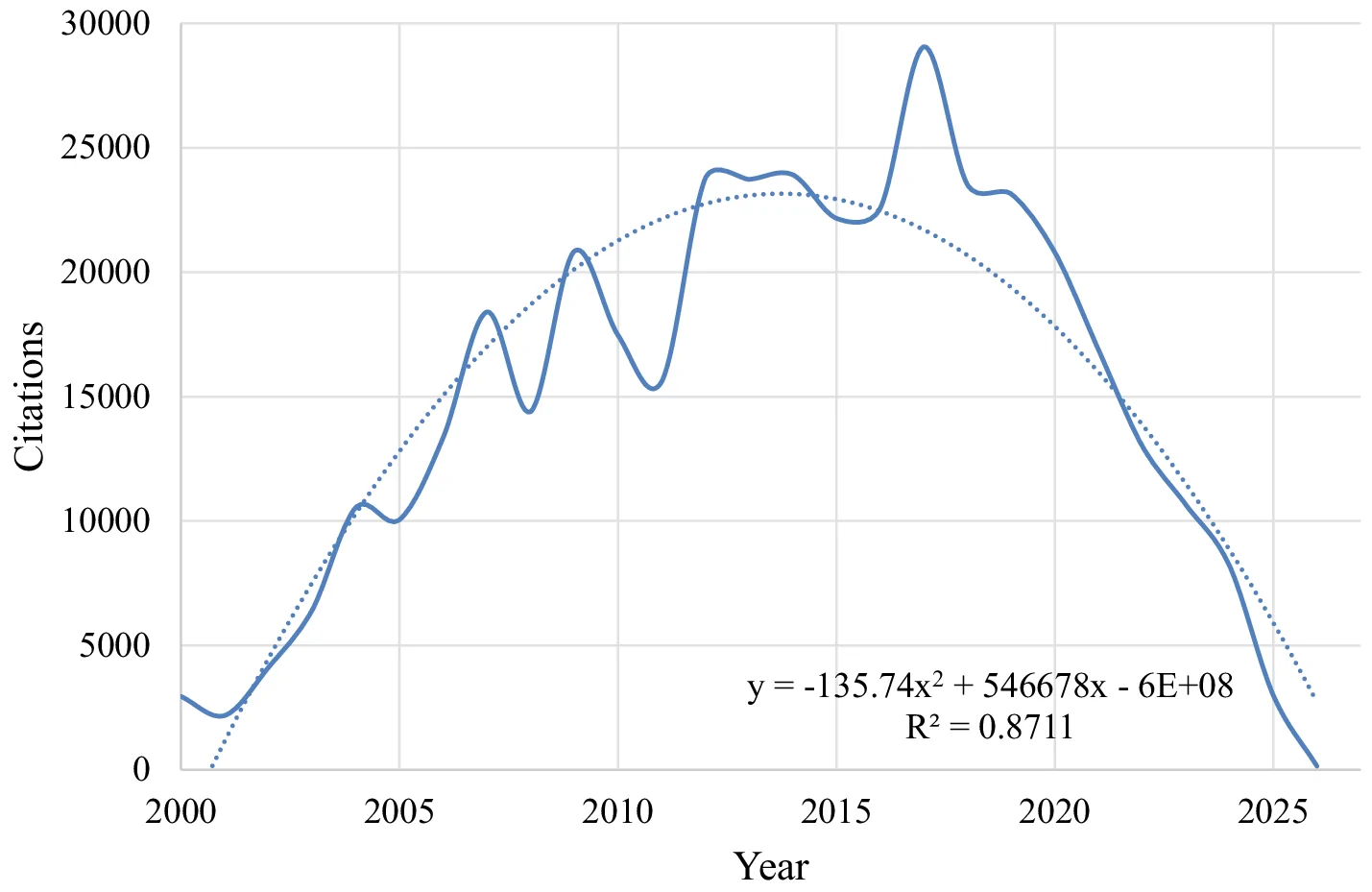
Dynamics of publication citation rates by year.

An analysis of total citations of publications confirmed the significant role of works published in 2003–2007 and the attainment of peak scientific impact by 2017. The decline in indicators in 2024–2026 is due to the natural time lag required for new publications to be indexed and accumulate citations (Figure 7).

**Figure 8:**
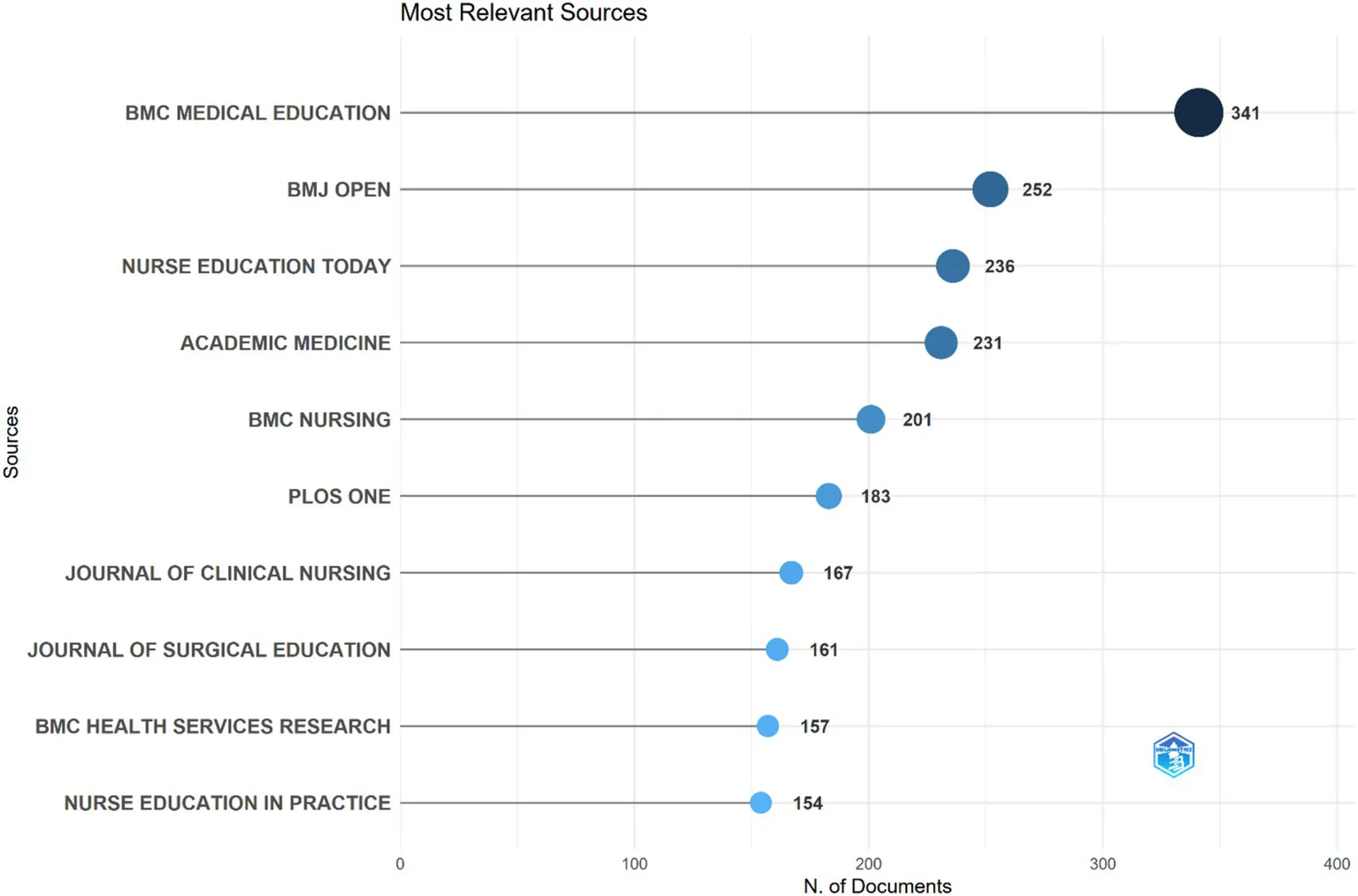
Top 10 most productive journals.

The publications were distributed across 3,361 academic journals, covering various areas of medical and related sciences. A significant proportion of the articles were published in leading specialist journals focusing on healthcare professional education, quality of care, and patient safety (Figure 8).

**Figure 9:**
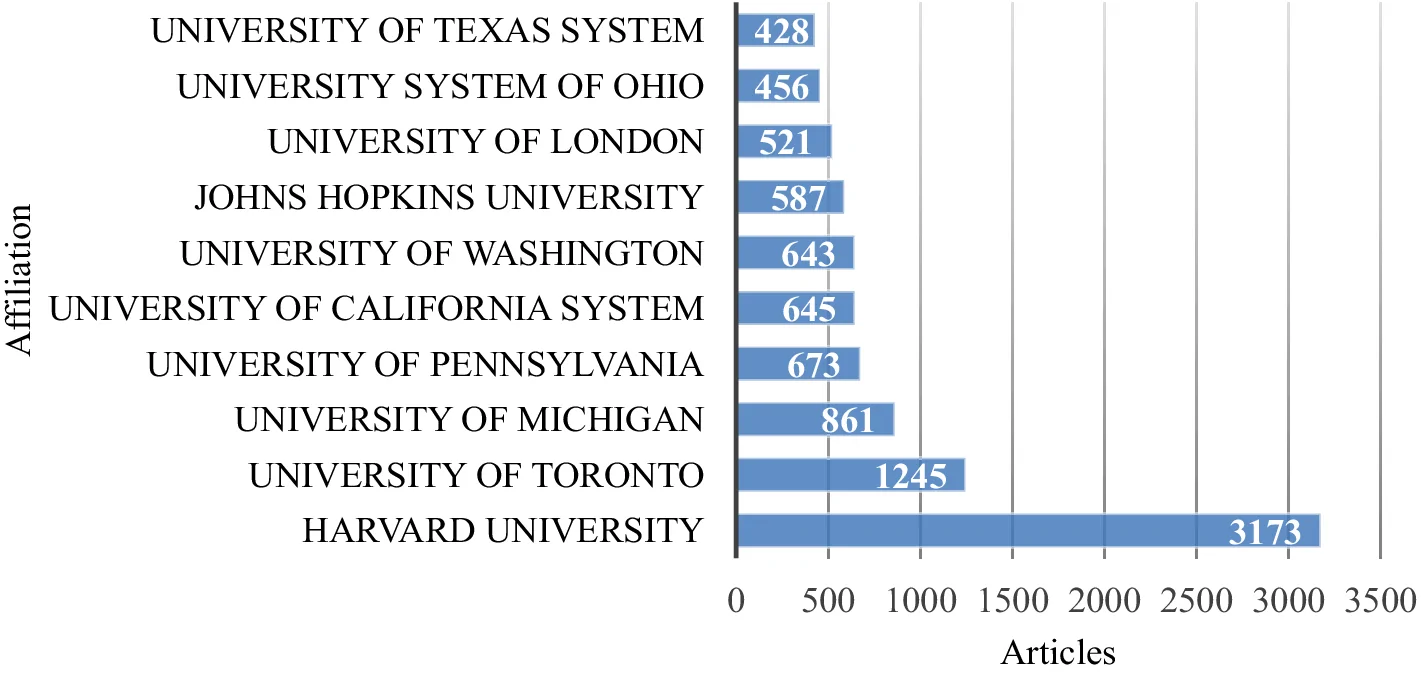
Top 10 leading research institutions.

An institutional analysis has revealed the dominance of major academic centers, with Harvard University, the University of Toronto, the University of Michigan, the University of Pennsylvania, the University of California system and the University of Washington occupying the leading positions. The results show that the largest number of publications come from large university systems in North America (Figure 9).

**Figure 10:**
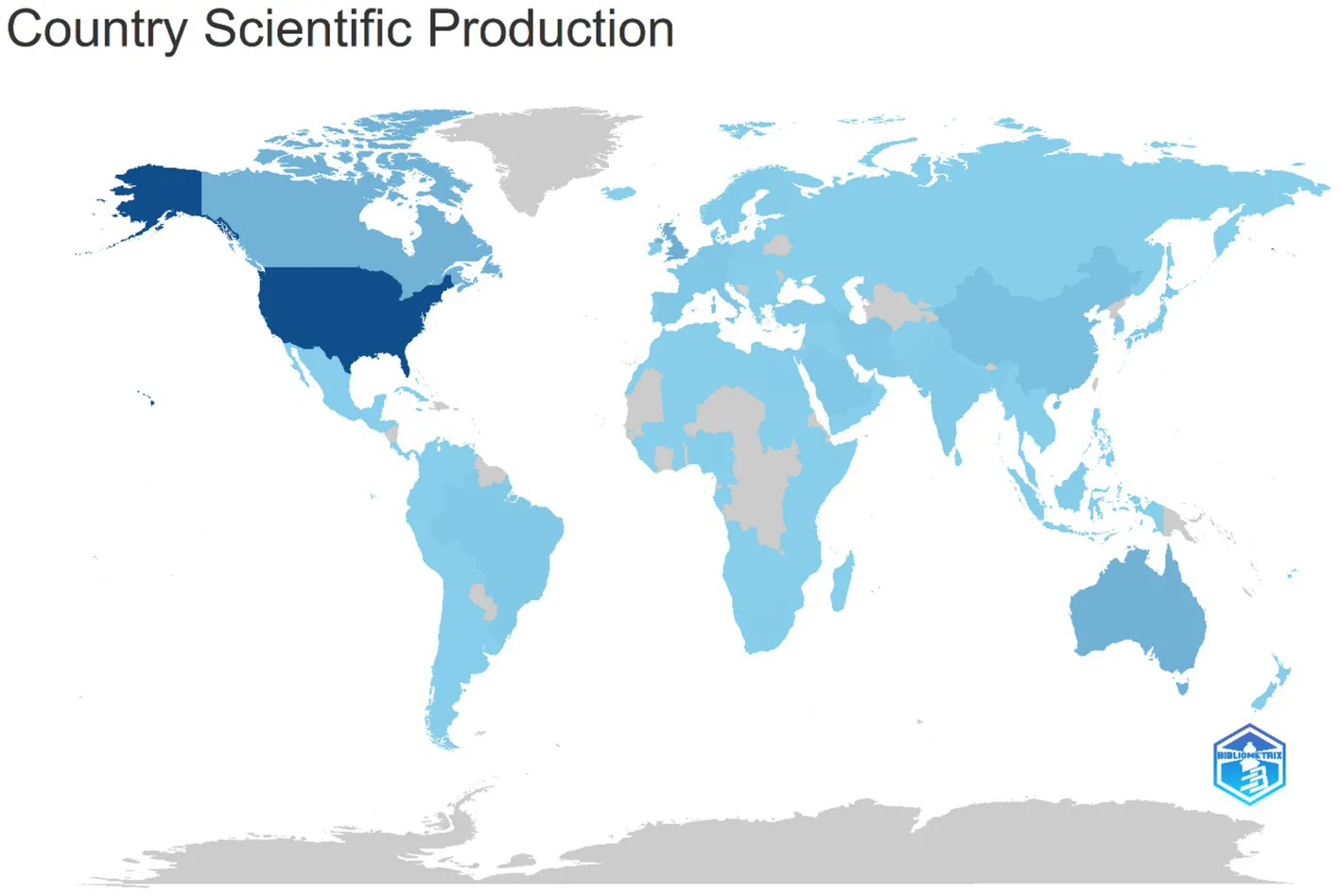
Geographical distribution of research productivity. The shades of blue on the map reflect the volume of scientific publications by country, based on the analyzed dataset. The darker the shade, the higher the number of papers identified. Grey indicates countries for which there are no publications on this topic in the final sample. This visualization reflects solely the density of the publication flow relevant to the search strategy under investigation.

A geographical analysis demonstrated the global scope of developments in this field, covering 142 countries. The United States held the leading position, followed by the United Kingdom, Australia, Canada, and China (Figure 10).

**Figure 11:**
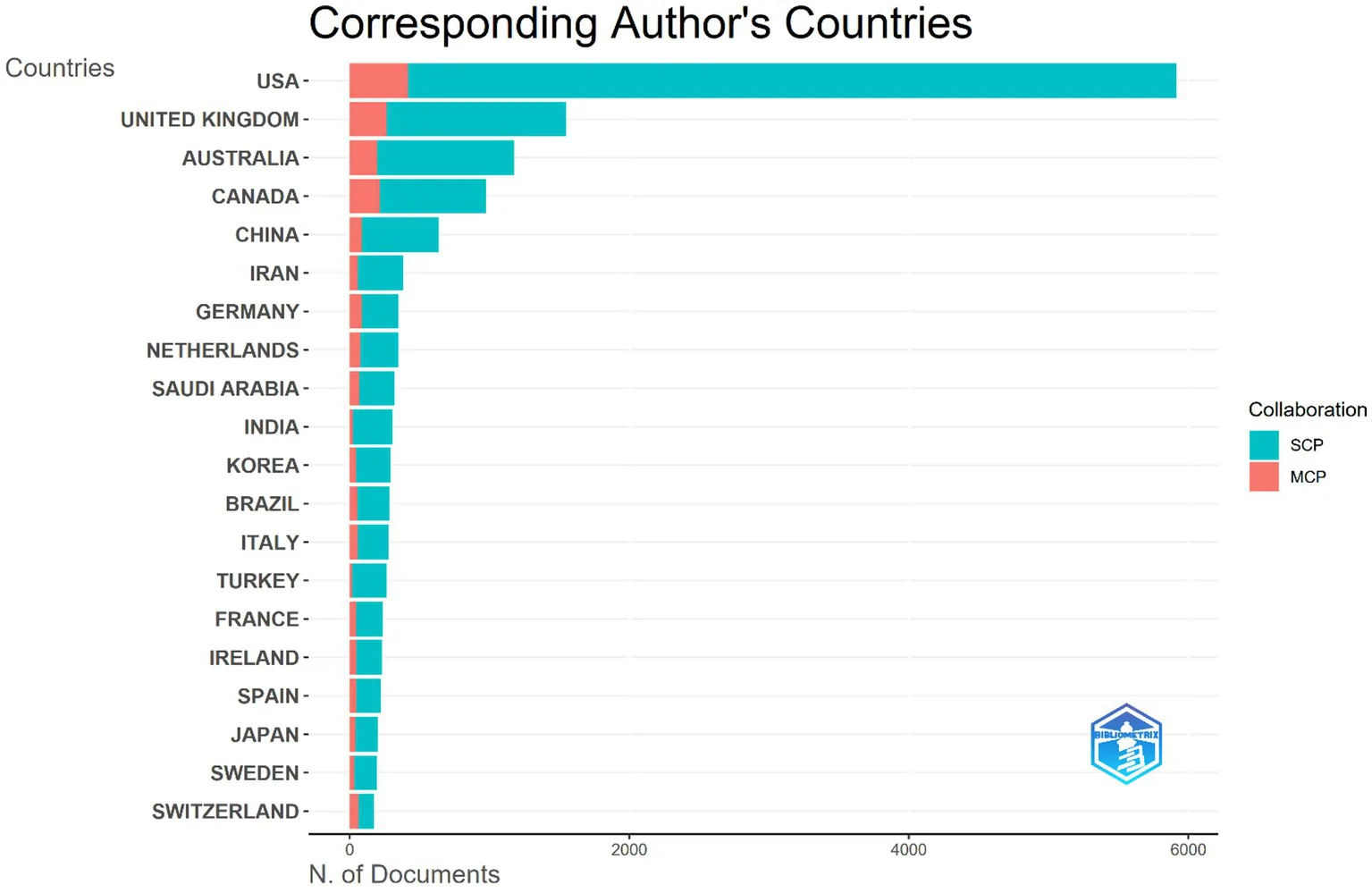
International collaboration and the Single Country Publications/Multiple Country Publications (SCP/MCP) structure. The color turquoise (SCP, Single Country Publications) indicates the proportion of articles written by researchers from a single country; the color red (MCP, Multiple Country Publications) indicates the proportion of internationally co-authored works. The analysis took into account the ratio of these types for the countries under review, which made it possible to reflect the internal structure of their national portfolios regardless of the absolute volume of publications.

An analysis of corresponding authors’ countries showed that the United States maintained a dominant position in terms of total publication volume. However, in terms of the proportion of international cooperation within their national portfolios, the Netherlands (25.14%), Germany (25.07%) and Canada (22.05%) lead the way, whilst for the US this figure stands at 7.05% (Figure 11).

Overall, there has been an increase in publishing activity, a broadening of the geographical scope of research, and a rise in international collaboration.

### 3.2 Temporal trends in keywords and citation impact

#### 3.2.1 2000–2008: keyword bursts and most cited publications

Figure 2 shows the evolution of keyword bursts during 2000–2008 (Figure 2). In the early stages, systemic and conceptual keywords predominated, including “healthcare and public health,” “diagnostic error,” and “professional competence”. From 2003, the thematic scope expanded to include keywords related to “medical education,” “prevention,” “standardization,” and clinical practice. By 2006–2008, the keyword “patient safety” had become one of the most frequently used terms, with increased use of keywords related to professional competence, communication, and empathy.

The most cited publications of this period focused on the systemic causes of medical errors, the influence of psychological factors on clinical practice, and educational strategies to improve the quality of healthcare. Among them were studies by Koppel R. (23) on the risks of computerized physician order entry systems (*n* = 1,408 citations), West C. P. et al. (24) on the association between self-perceived medical errors and the emotional well-being of residents (*n* = 1,389 citations), Reeves S. et al. (25) on interprofessional education (*n* = 767 citations), and Cronenwett L. et al. (26), who developed the concept of quality and safety education for nurses (*n* = 766 citations).

#### 3.2.2 2009–2014: keyword bursts and most cited publications

Between 2009 and 2014, there was a rapid expansion in the range of keywords describing patient safety research (Figure 3).

In the early stage, key bursts were associated with error prevention systems, medication safety, decision-support systems, quality of care, and legal aspects. In subsequent years, keywords related to evidence-based education, nursing, controlled studies, and assessment tools became more frequent. By 2011–2014, “patient safety” (term frequency = 1,041) was among the most frequently occurring keywords, accompanied by more frequent occurrence of terms such as quality improvement, professionalism, standards, and psychology.

The most cited publications of this period focused on physician burnout, simulation-based education, interprofessional collaboration, and systemic models of safety. A highly influential study by Shanafelt T. D. et al. (27) addressed the high prevalence of professional burnout among physicians and its potential impact on the quality of healthcare delivery (*n* = 2,468 citations). High citation counts were also observed for the works of Okuda Y. et al. (12) (*n* = 825 citations) and Motola I. et al. (28) (741 citations), demonstrating the effectiveness of simulation-based education. In addition, studies by Manser T. (29) and Holden R. J. (30) emphasized the role of teamwork and human factors frameworks in improving patient safety.

#### 3.2.3 2015–2018: keyword bursts and most cited publications

Figure 4 shows further diversification of research keywords during 2015–2018.

In the early stage, key bursts were associated with “emergency health services,” “high-fidelity simulation,” “self-evaluation,” “communication,” and “adverse events,” reflecting growing interest in simulation-based education, the development of communication skills, self-assessment of professional training, and the prevention of adverse events.

In 2016–2018, patient safety (1,213 term frequency) maintained its dominant position, accompanied by an increase in the terms such as education, health education, framework, communication, adverse events, and quality improvement.

The most cited publications of this period were primarily focused on the professional well-being of healthcare workers, workforce policy, and the quality of medical training. A highly influential study by Shanafelt T. D. et al. (31) addressed organizational strategies for preventing physician burnout and their importance for the quality of healthcare delivery (*n* = 1,249 citations). Significant contributions were also made by Jones T. L. et al. (32), who systematized evidence on missed nursing care (*n* = 550 citations), and Aiken L. H. et al. (33), who demonstrated the impact of nursing skill mix on the quality of care and clinical outcomes (*n* = 498 citations).

#### 3.2.4 2019–2026: keyword bursts and most cited publications

During 2019–2026, the most frequently occurring keywords were related to healthcare professional education, patient safety, digital healthcare, organizational sciences, and artificial intelligence (Figure 5). The most pronounced bursts were observed for the keywords “patient safety” (2,885), “education” (1,751), “medical education” (863), “medical errors” (256), and “drug safety” (149). Additional keywords related to demographic characteristics of the study population and the context of healthcare delivery, including “female,” “male,” “middle-aged,” and “age factors,” were also identified. From 2024–2026, the keywords “artificial intelligence” and “generative artificial intelligence” appeared among the emerging research topics. Keywords related to organizational management, practice, systematic review, and scoping review also became more frequent during this period.

The most cited publications of this period reflected the development of innovative educational technologies, the digitalization of medical training, and the enhancement of competencies in patient safety. A highly influential study by Elendu C. et al. (4) demonstrated the potential of simulation-based training, including virtual reality and high-fidelity mannequins, in improving clinical training and reducing errors (*n* = 446 citations). Significant contributions were also made by Gentry S. V. et al. (34), who highlighted the role of serious gaming and gamification in the education of healthcare professionals (*n* = 384 citations), as well as Konttila J. et al. (35), who emphasized the need to develop digital, communication, ethical, and organizational competencies in the context of healthcare digitalization (*n* = 283 citations). The authors note that the use of interactive and digital educational technologies improves the quality of training of specialists (34, 35), which, in turn, contributes to the creation of a safer clinical environment.

### 3.3 Cluster analysis of keywords

**Figure 12:**
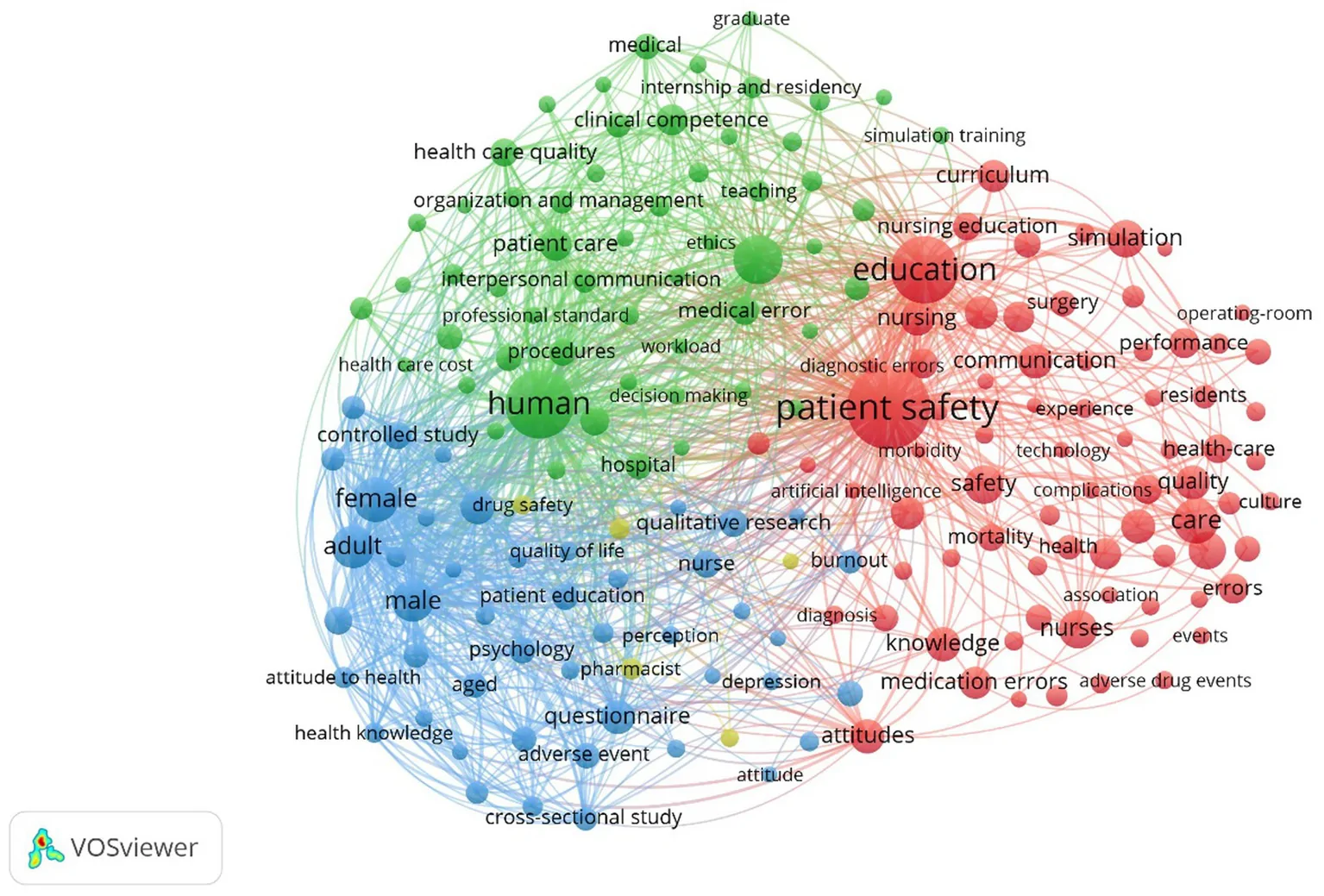
Keyword co-occurrence and thematic clusters in research on medical errors, patient safety, and medical education (Web of Science, Scopus, 2000–2026). The network map showing the co-occurrence of keywords was generated using VOSviewer. The size of the nodes is proportional to the overall frequency of the keywords, whilst their proximity to one another reflects the strength of their co-occurrence in the titles and abstracts of articles within the search strategy.

A cluster analysis of keyword co-occurrences, carried out using VOSviewer, identified four thematic clusters reflecting the structure of the research field of healthcare professional education, medical errors, and patient safety (Figure 12).

The “Educational strategies and competency development” cluster (red) is the largest and includes keywords related to educational approaches and the development of professional competencies (education, curriculum, medical education, simulation, interprofessional education, competence, skills). The cluster also incorporates terms related to clinical reasoning and decision-making (diagnostic error, decision-making), as well as the assessment of educational outcomes (outcomes, performance).

The “Healthcare systems and quality management” cluster (green) includes terms characterizing the organizational and institutional level (health care system, health care policy, standards, risk management, safety management, quality of health care). This cluster reflects research focused on the development of quality management systems and ensuring patient safety at the level of healthcare organizations.

The “Human factors and epidemiology of errors” cluster (blue) brings together keywords related to the psychological and organizational aspects of medical practice, including healthcare worker burnout (burnout), fatigue, anxiety, and mental health, as well as epidemiological characteristics (prevalence, risk factor, epidemiology) and research methodologies (surveys, qualitative research). This cluster reflects a focus on behavioral and professional determinants of patient safety.

The “Medication safety” cluster (yellow) represents a specialized applied domain and includes the terms drug safety, pharmacist, pharmacy, and prescription, which are related to the prevention of medication errors and the assurance of safe pharmacotherapy.

Taken together, the identified clusters reflect a multi-level structure of the research field, encompassing the educational (micro), organizational (macro), and behavioral (meso) levels, as well as a distinct specialized area focused on medication safety.

To facilitate the interpretation of the identified thematic clusters, representative keywords characterizing each cluster are provided in Supplementary Table S2.

## 4 Discussion

This bibliometric analysis demonstrates that the research field relating to healthcare professional education, medical errors, and patient safety is characterized by increasing publication activity, expanding international collaboration, and thematic diversity of research. An analysis of the current literature reveals that studies of educational interventions primarily focus on educational outcomes, while assessments of their impact on patient safety indicators are significantly less common. The identified bibliometric trends indicate growing interest in this topic within the scientific community and create the preconditions for further research aimed at examining the relationship between educational interventions and clinical outcomes.

In contrast to previously published bibliometric studies, which have predominantly focused on individual aspects (for example, simulation-based education or patient safety training) (13–15), the present study integrates healthcare professional education, medical errors, and patient safety within a unified analytical framework. This approach allows us to identify the relationships between the main thematic areas of research, reflecting individual competencies (micro level), the human factor (meso level) and organizational systems (macro level).

The four identified stages of the research field’s development reflect a progressive transition from the conceptualization of medical errors to the development of integrated systemic and technological solutions. The findings should be considered in the context of the WHO Global Action Plan for Patient Safety 2021–2030. The high proportion of studies on healthcare worker training and quality management reflects the growing attention of the scientific community to these areas, which are among the key priorities for ensuring patient safety.

However, the geographic concentration of scientific productivity in high-income countries highlights the importance of expanding future research, educational, and practical initiatives.

### 4.1 Educational strategies and competency development

The presence of the educational cluster reflects the high interest of the scientific community in the training of medical professionals in the field of patient safety. The evolution of keywords reflects a shift from traditional knowledge transmission toward the development of comprehensive competencies, including clinical reasoning, teamwork, and a safety culture. Simulation-based learning, interprofessional education, and digital educational technologies are among the most actively studied areas of modern medical education (4, 5). Their widespread representation in the scientific literature demonstrates the significant importance of these approaches to the research community.

However, according to published research, the effectiveness of healthcare professionals’ training to improve patient safety is predominantly evaluated indirectly, through participants’ self-assessments, rather than through objective clinical outcomes. Thus, in a review of interprofessional educational interventions, Jiang Y et al. note that the assessment of outcomes in most studies is based on participants’ self-assessment and knowledge measurement, rather than on objective clinical indicators (36). Similarly, Sung T.-C. et al., analyzing a simulation-based interprofessional program in an intensive care unit, note the greatest progress in the areas of communication and leadership, but these do not always lead to a significant reduction in errors and require further refinement (6).

Systematic review points to high variability in educational programs and a lack of standardized assessment methods, which limits the comparability of results and complicates the interpretation of their clinical significance (37). The findings highlight the relevance of further research aimed at assessing the impact of educational interventions on patient safety outcomes.

An important component of training is the development of systematic clinical thinking, as evidenced by the grouping of concepts such as decision-making, diagnostic error and competence within a single thematic cluster. Thus, research by Mcloughlin C. et al. demonstrates that inadequate training of doctors can lead to iatrogenic harm, which strengthens the argument in favor of integrating clinical reasoning and communication into educational programs (38).

An analysis of keyword trends reveals a rapid increase in the volume of scientific literature devoted to the potential of innovative technologies in the training of healthcare professionals. However, this growth is outpacing the accumulation of long-term empirical data on their actual impact on clinical outcomes and the reduction in the incidence of medical errors.

Virtual and augmented reality technologies (39–41) demonstrate a positive impact on learning; however, their association with clinical outcomes remains limited. Similarly, virtual patients (42), social media (43), and distance learning technologies (44) improve learner engagement but require further investigation.

Particular attention is drawn to the growing interest in artificial intelligence and generative models, which are shaping a new area of research (45). The rise in mentions of the keywords “artificial intelligence” and “generative artificial intelligence” between 2024 and 2026 reflects high expectations regarding the potential of these technologies in healthcare professional education and clinical decision-making support.

These technologies have significant potential to transform healthcare professional education and clinical practice, including decision support and the personalization of learning. Moreover, a review by Aydin S. et al. shows that large language models can make medical information more accessible to patients; however, issues of accuracy, readability, and ethical risks remain unresolved (46). Their implementation is also accompanied by a number of outstanding concerns, including algorithmic transparency, potential systematic biases, ethical considerations, and patient safety. In addition, there is a risk that AI tools may influence clinical reasoning, which requires further investigation. Therefore, prospective studies are needed to assess not only educational effectiveness but also the long-term impact of these technologies on clinical practice.

Among innovative approaches, cinemeducation should be noted. Cambra-Badii I. et al. demonstrated that the use of film excerpts enables medical students to more deeply analyze system errors and human factors compared to traditional teaching methods (47). Such approaches may complement educational curricula.

A review by Portela Dos Santos O. et al. shows that the most effective formats are blended educational approaches incorporating mentorship components, as well as digital and online learning formats (48).

### 4.2 Human factors and epidemiology of errors

The identification of a cluster related to human factors highlights the important role of psychological and behavioral aspects in ensuring patient safety. Systematic reviews and meta-analyses demonstrate a consistent link between burnout among healthcare professionals and an increase in the frequency of medical errors and adverse events, as well as a decline in the quality of care. Thus, a systematic review by Hall L. H. et al., covering a wide range of medical specialties, found that low levels of healthcare worker well-being and high levels of burnout are associated with impaired patient safety in most studies, although causal relationships require confirmation in prospective research (49). A meta-analysis by Li L. Z. et al., including 85 studies with more than 288,000 nurses, showed that burnout is significantly associated with increased rates of medical errors, missed nursing care, patient falls, and other adverse events (50). A large national study by Tawfik D. S. et al. found that physicians with signs of emotional exhaustion are significantly more likely to report serious medical errors (51). Similarly, Melnyk B. M. et al. demonstrated the same pattern among intensive care unit nurses, emphasizing that a supportive work environment contributes to a reduction in preventable errors (52).

Despite this, the integration of human factors into educational programs remains limited. Of particular significance is the phenomenon of the “second victim,” reflecting the psychological impact of medical errors on healthcare workers. Thus, Mira J. J. et al. found in a systematic review that medical students who witness or are involved in medical errors experience a significant psychological impact characteristic of “second victim” syndrome; however, support and educational programs for this group are rare (53). Sánchez-García A. et al., in an observational study of European medical and nursing faculties, demonstrated that the “second victim” phenomenon is the most frequently overlooked topic in curricula, despite awareness of its significance (54). A qualitative study by Srulovici E. et al. confirmed that the main barriers to including this topic in educational programs are overloaded curricula, insufficient teacher training, and strict regulatory requirements (55). Despite mounting evidence of its prevalence and significance, this topic remains under-represented in educational programs.

Bibliometric data indicate that the literature on mental health, resilience, and error management in the context of patient safety remains heterogeneous. Mental health and burnout prevention have been actively integrated into healthcare curricula in many countries over the past decade. However, the WHO Global Action Plan on Patient Safety 2021–2030 calls for further progress toward access to mental health services and social support for healthcare workers, including work-life balance counseling, as well as risk assessment and mitigation, to combat burnout, improve well-being, and develop resilience (1). These objectives are particularly relevant for healthcare systems facing severe staffing shortages, the consequences of emergencies or wars, and humanitarian crises, which are fundamentally changing the working conditions of healthcare personnel. Future work should focus on strengthening the implementation and evaluation of existing curricula, including their impact on healthcare worker well-being, retention, safety culture, and clinically relevant patient safety indicators.

### 4.3 Healthcare systems and quality management

The cluster relating to healthcare systems reflects a shift toward proactive safety management models, encompassing standardization, risk management, and the development of a safety culture.

However, significant barriers remain, including hierarchical structures, poor communication, and fragmentation between educational and clinical processes (56). The substantial variability in patient safety training programs noted in the review by Aldardeir N. et al. indicates a lack of uniform training standards in this field (57). This highlights the need to develop standardized yet adaptable training models that take into account the specific characteristics of different healthcare systems, as well as to enhance the skills of teaching staff.

### 4.4 Medication safety

Medication safety represents a distinct research cluster, encompassing publications on educational approaches and the prevention of medication prescribing errors. For instance, studies by Jaam M. et al. (58) and Martin P. et al. (59) demonstrate a significant reduction in the incidence of incorrect medication prescribing when educational programs delivered by pharmacists are used.

Modern educational approaches, including the SafeMedicate platform and online courses on safe prescribing (60), contribute to improving competencies in the field of safe prescribing. Similarly, research by Graham E. B. et al. highlights the effectiveness of distance learning in clinical pharmacology (61). These findings point to the significant role of educational strategies in ensuring medication safety.

## 5 Conclusion

This study makes a significant contribution to the field by integrating healthcare professional education, medical errors, and patient safety within a single analytical framework. Unlike previous bibliometric studies, which focused on individual aspects, this analysis has identified interrelationships between educational, behavioral, and organizational factors and proposed a four-phase model for the development of the research field.

Interpreting thematic clusters from a multi-level (micro-meso-macro) perspective allowed us to characterize the structure of the research field and identify key areas of development. The findings reflect a growing interest in medical education, patient safety, and organizational aspects of healthcare.

The study’s results demonstrate a steady increase in publication activity, an expansion of thematic areas, and a strengthening of the interdisciplinary nature of research in the fields of medical education, medical errors, and patient safety. Bibliometric analysis revealed growing scientific interest in educational strategies and patient safety issues, reflecting the scientific community’s focus on priority areas in this field. However, the relationship between educational interventions and patient safety outcomes requires further study using appropriate methodological approaches.

The strength of this study is its integrated bibliometric framing of healthcare professional education, medical errors, and patient safety across two major citation databases. This approach enabled simultaneous assessment of publication growth, influential sources, geographical and institutional contributions, temporal keyword trends, and thematic clusters. The analysis therefore extends prior bibliometric work that focused more narrowly on patient safety education, nursing student education, or simulation-based medical education.

This study has several limitations. The use of the Web of Science and Scopus databases ensures broad coverage of publications; however, it may lead to the underrepresentation of regional studies. The restriction to English-language publications also reduces the global representativeness of the findings. In addition, the results of keyword clustering depend on the selected threshold values and algorithms, which may influence the interpretation of thematic structures. In line with the used methodology, it should be noted that automatic bibliometric mapping inevitably includes publications that examine patient safety education within the context of broader interdisciplinary concepts. This is related to the use of a broad search strategy, which is characteristic of bibliometric studies and may lead to the inclusion of publications that are only partially related to the research topic. To assess the potential impact of this limitation, a corpus specificity audit was conducted. The audit results showed that the majority of reviewed records were consistent with the research topic, although some publications had a broader or indirect focus and only partially reflected the core concepts of the study. Therefore, the findings should be interpreted as reflecting general scientific trends within a broad bibliometric corpus rather than as an exhaustive sample of individual educational interventions.

The obtained results, combined with an analysis of the current literature, allow us to identify several promising areas for further research. These include the development of clinically relevant indicators linking educational interventions with patient safety outcomes, as well as the study of educational and organizational approaches aimed at supporting the mental health, well-being, and preventing burnout in healthcare workers, including issues of their implementation and evaluation, and the potential of emerging technologies, particularly artificial intelligence, in medical education and patient safety. These areas are particularly relevant for healthcare systems operating in conditions of staff shortages, emergencies, and humanitarian crises. The identified thematic areas reflect current trends in patient safety research and cover a number of issues outlined in the WHO Global Action Plan for Patient Safety 2021–2030, including developing patient safety competencies, interprofessional education, and supporting the well-being of healthcare workers.

## Data Availability

The original contributions presented in the study are included in the article/Supplementary material, further inquiries can be directed to the corresponding authors.
