# Supplementary material for: Global trends and thematic clusters in healthcare professional education, medical errors, and patient safety: a bibliometric analysis, 2000–2026
Source: Front Med (Lausanne). 2026 Sep 3;13:1892822. doi: 10.3389/fmed.2026.1892822 (PMC13581705; doi:10.3389/fmed.2026.1892822)
Supplement: Supplementary file 1 [file Supplementary_file_1.docx]

*Supplementary information*

**Global Trends and Thematic Clusters in Healthcare Professional Education, Medical Errors and Patient Safety: A Bibliometric Analysis, 2000-2026**

**Birgit Hladschik-Kermer^1^, Olena Litvinova^2,3,*^, Michel-Edwar Mickael^4^, Maima Matin^4^, Ahmed Fatimi^5^, Anass Belbachir^6,7^, Mairim Russo Serafini^8,9^, Maria Kletecka-Pulker^2,10^, Atanas G. Atanasov^2,4,11*^, Thomas Wochele-Thoma^2,*^**

^1^Unit Medical Psychology, Department of Public Mental Health, Center for Public Health, Medical University of Vienna, 1090 Vienna, Austria

^2^Ludwig Boltzmann Institute Digital Health and Patient Safety, Medical University of Vienna, Vienna, Austria

^3^Department of Management, Marketing and Quality Assurance in Pharmacy, National University of Pharmacy of the Ministry of Health of Ukraine, Kharkiv, Ukraine

^4^Department of Biotechnology and Nutrigenomics, Institute of Genetics and Animal Biotechnology of the Polish Academy of Sciences, Magdalenka, Poland.

^5^Chemical Science and Engineering Research Team, Polydisciplinary Faculty of Beni Mellal, Sultan Moulay Slimane University, P.O. Box 592 Mghila Campus, Beni Mellal 23000, Morocco

^6^Regenerative Medicine Center, Mohammed VI University Hospital, Marrakech

^7^Faculty of Medicine and Pharmacy, Cadi Ayyad University, Marrakech, Morocco

^8^Postgraduate Program in Health Sciences, Federal University of Sergipe, Aracaju, Sergipe, Brazil

^9^Department of Pharmacy, Federal University of Sergipe, São Cristovão, Sergipe, Brazil

^10^Institute for Ethics and Law in Medicine, University of Vienna, Vienna, Austria

^11^Department of Biochemistry, Saveetha Medical College and Hospital, Saveetha Institute of Medical and Technical Sciences, Chennai, Tamil Nadu, India

*** Correspondence:**
Olena Litvinova
[hlitvinova@gmail.com](mailto:hlitvinova@gmail.com)

Atanas G. Atanasov
[a.atanasov@igbzpan.pl](mailto:a.atanasov@igbzpan.pl)

Thomas Wochele-Thoma
[Thomas.Wochele-Thoma@caritas-wien.at](mailto:Thomas.Wochele-Thoma@caritas-wien.at)

**Supplementary Table S1. Search strategy**

| Parameter | Web of Science | Scopus |
| --- | --- | --- |
| Search field | Topic (TS) | TITLE-ABS-KEY |
| #1 | TS = (“teaching*” OR “curriculum*” OR “curricula*” OR “educat*” OR “workshop*” OR “seminar*” OR “online course*” OR “eLearning*” OR “online learning*” OR “E-learning*” OR “electronic learning*” OR “computer-assisted learning*” OR “computer-mediated learning*” OR “computer-based learning*” OR “digital learning*” OR “simulation-based training*” OR “simulation-based learning*”) | TITLE-ABS-KEY = (“teaching*” OR “curriculum*” OR “curricula*” OR “educat*” OR “workshop*” OR “seminar*” OR “online course*” OR “eLearning*” OR “online learning*” OR “E-learning*” OR “electronic learning*” OR “computer-assisted learning*” OR “computer-mediated learning*” OR “computer-based learning*” OR “digital learning*” OR “simulation-based training*” OR “simulation-based learning*”) |
| #2 | TS = (“medical error*” OR “medication error*” OR “clinical error*” OR “diagnostic error*” OR “adverse event*” OR “adverse outcome*” OR “patient safety*” OR “patient harm*” OR “clinical incident*” OR “preventable harm*” OR “iatrogenic*”) | TITLE-ABS-KEY = (“medical error*” OR “medication error*” OR “clinical error*” OR “diagnostic error*” OR “adverse event*” OR “adverse outcome*” OR “patient safety*” OR “patient harm*” OR “clinical incident*” OR “preventable harm*” OR “iatrogenic*”) |
| #3 | TS = (“student*” OR “resident*” OR “trainee*” OR “physician*” OR “doctor*” OR “nurse*” OR “clinician*” OR “healthcare professional*”) | TITLE-ABS-KEY = (“student*” OR “resident*” OR “trainee*” OR “physician*” OR “doctor*” OR “nurse*” OR “clinician*” OR “healthcare professional*”) |
| #4 | #1 AND #2 AND #3 AND DATE=2000-2026 (as of April 27, 2026)  **Records retrieved: 14,872** | #1 AND #2 AND #3 AND DATE=2000-2026 (as of April 27, 2026)  **Records retrieved: 9,101** |
| #5 | #4 AND Filters applied: English language; Articles and Reviews  **Records retrieved: 13,502** | #4 AND Filters applied: English language; Articles and Reviews  **Records retrieved: 7,395** |
| Export format | Plain Text | BibTeX |
| Total result identified | **20,897** | |
| Duplicate records removed | **3,440** | |
| Final merged dataset after duplicate removal | **17,457** | |

**Supplementary Table S2. Representative keywords defining the thematic clusters identified by the keyword co-occurrence analysis**

| Cluster | Title | Representative keywords |
| --- | --- | --- |
| Red cluster | Educational strategies and competency development | Patient safety; education; curriculum; communication; competence; simulation; teamwork; decision-making; diagnostic error; medical education |
| Green cluster | Healthcare systems and quality management | Health care quality; health care system; health care delivery; patient care; clinical competence; continuing education; education program; organization and management; professional competence; leadership |
| Blue cluster | Human factors and epidemiology of errors | Burnout; epidemiology; fatigue; awareness; health personnel; attitude of health personnel; mental health; prevalence; qualitative research; risk factors |
| Yellow cluster | Medication safety | Drug safety; pharmacist; pharmacists; pharmacy; prescription |

**Note.** Representative keywords are selected from the keywords assigned to each cluster to illustrate its principal thematic focus. The table is intended to facilitate interpretation of the network visualization and does not represent the complete list of keywords within each cluster.
